# Badminton Players’ Expert Advantage in Anticipation Based on Visual and Auditory Cues: Evidence from the Drift Diffusion Model

**DOI:** 10.3390/bs16071203

**Published:** 2026-07-16

**Authors:** Senlin Lan, Xu Shi, Ya Gao, Guanchen Zhou, Zhihua Yang, Chuntian Wang, Ye Mao, Xiaotong Wang, Jialian Cao, Haoping Yang

**Affiliations:** 1 School of Competitive Sports, Beijing Sport University, Beijing 100084, China; 2024240577@bsu.edu.cn (S.L.);; 2 Department of Physical Education, School of Law and Humanities, Beijing University of Chemical Technology, Beijing 100084, China; 3 School of Psychology, Beijing Sport University, Beijing 100084, China; 4 School of Physical Education, South China University of Technology, Guangzhou 510641, China

**Keywords:** badminton, anticipation, perceptual–cognitive expertise, visual cues, auditory cues, hierarchical drift diffusion model, decision-making

## Abstract

Anticipation is central to perceptual–cognitive expertise in badminton, but the decision mechanisms through which experts use visual and auditory cues remain unclear. This study examined expert–novice differences in unimodal anticipation and latent decision processes estimated by a hierarchical drift diffusion model (HDDM). Sixty-six participants (33 experts and 33 novices) completed a landing-region anticipation task under visual-only and auditory-only conditions. Behavioral performance was analyzed using mixed analyses of variance (ANOVAs), and accuracy and reaction time were jointly modeled with HDDM parameters: boundary separation (*a*), drift rate (*v*), non-decision time (*t*), and starting-point bias (z). Experts showed higher overall accuracy than novices, *F*(1, 64) = 56.46, *p* < 0.001, *ηp*^2^ = 0.469, whereas reaction time did not differ significantly by group. The auditory-only condition produced higher accuracy, *F*(1, 64) = 12.69, *p* = 0.001, *ηp*^2^ = 0.165, but longer reaction times, *F*(1, 64) = 150.32, *p* < 0.001, *ηp*^2^ = 0.701. HDDM results showed higher drift rates in experts under both visual-only (BF10 = 1992.845) and auditory-only (BF10 = 24.354) conditions; auditory-only anticipation also involved higher boundary separation (BF10 = 53.095). These findings suggest that badminton expertise is associated with higher anticipatory accuracy and more efficient evidence accumulation under unimodal cue conditions, while auditory-only anticipation may also involve a more cautious decision threshold.

## 1. Introduction

Sport anticipation is a fundamental basis for high-level performance in open-skill sports. In sports characterized by high time pressure and intense opposition, such as badminton and tennis, athletes often cannot wait until complete ball-flight information becomes available before initiating a response. Instead, they must predict the outcome and prepare their actions in advance under conditions of incomplete information, relying on opponents’ movements and environmental cues. Accordingly, anticipatory ability is widely regarded as a core component of sport-specific perceptual–cognitive expertise and a key marker distinguishing athletes of different skill levels (Loffing & Cañal-Bruland, 2017; Müller & Abernethy, 2012; Song et al., 2025; Huesmann & Loffing, 2024).

Badminton places particularly high demands on this ability. Owing to the high speed of the shuttlecock, the compact rhythm of rallies, and the wide variety of stroke techniques, badminton players usually have only a very brief time window in which to use opponents’ hitting actions, racket trajectories, and transient information before and after shuttle contact to infer the direction and landing region of the incoming shuttle. Previous studies have shown stable differences between individuals with different expertise levels in badminton anticipation tasks. Moreover, these differences are not entirely consistent across different task contexts, such as rally strokes and serves, suggesting that badminton anticipation is highly task-specific and context-sensitive (Robertson et al., 2022; De Waelle et al., 2023; Wu et al., 2026; X. Wang et al., 2025).

Among the sensory bases of sport anticipation, visual cues have long been considered dominant. Expert players can more effectively use body posture, stroke actions, kinematic changes around racket–shuttle contact, and contextual probability information, thereby forming outcome predictions at an earlier stage. Classic studies and reviews have indicated that, in striking sports, one important source of expert advantage lies in the efficient extraction and use of advanced visual cues (Abernethy & Russell, 1987; Abernethy et al., 2001; Müller & Abernethy, 2012; Gredin et al., 2023; D. Wang et al., 2025).

Auditory cues may also provide critical support for sport anticipation. The intensity and temporal structure of hitting sounds, as well as their congruence with action events, can provide athletes with additional information about hitting force, contact quality, and the subsequent trajectory of the incoming ball or shuttle. Previous studies have found that, in ball sports, early auditory information may sometimes be more beneficial than early visual information for making rapid judgments about stroke intensity or outcomes. In badminton and related racket sports, auditory information may also facilitate visuomotor response speed or enhance the perception of landing location (Sors et al., 2017; Hülsdünker et al., 2021; X. Wang et al., 2023, 2025).

Visual and auditory information differ in the types of information they provide, their temporal resolution, and the time windows within which they can be used. Vision is better suited to providing spatial structure and movement trajectory information, whereas audition can provide highly temporally precise cues at the moment an event occurs. For this reason, expert advantage may not emerge in exactly the same form across visual and auditory channels. In other words, differences between expert players and novice participants may not only be reflected in overall performance levels, but also in the processing mechanisms underlying different sensory channels (Loffing & Cañal-Bruland, 2017; Sors et al., 2017; X. Wang et al., 2023).

Existing research has provided substantial evidence for experts’ behavioral advantages in sport-related perceptual–cognitive tasks. Meta-analytic evidence suggests that experts typically show higher response accuracy, superior cue extraction ability, and more task-specific attentional allocation. In studies of naturalistic decision making in badminton, experts have also demonstrated more efficient contextual organization and action planning (Mann et al., 2007; Macquet & Fleurance, 2007). However, explanations of expert advantage in these studies have largely remained at the level of accumulated experience, attentional control, cue-utilization efficiency, and contextual knowledge. Executive functions have also been implicated in sport decision-making, particularly when athletes must regulate information processing and action selection under complex and uncertain conditions (Cao et al., 2025). Relatively few studies have further examined the latent decision-making mechanisms through which such advantages are realized. More importantly, although previous research has often examined visual-cue use or multisensory contexts, direct computational evidence comparing expert players and novice participants under separate visual-only and auditory-only conditions remains limited (Mann et al., 2007; Macquet & Fleurance, 2007; De Waelle et al., 2023). It remains unclear, however, whether expert advantages under separate visual-only and auditory-only conditions are driven mainly by evidence accumulation, decision thresholds, non-decision processes, or initial response bias (He et al., 2025; Weinberg et al., 2025; Yan et al., 2026).

**Figure 1:**
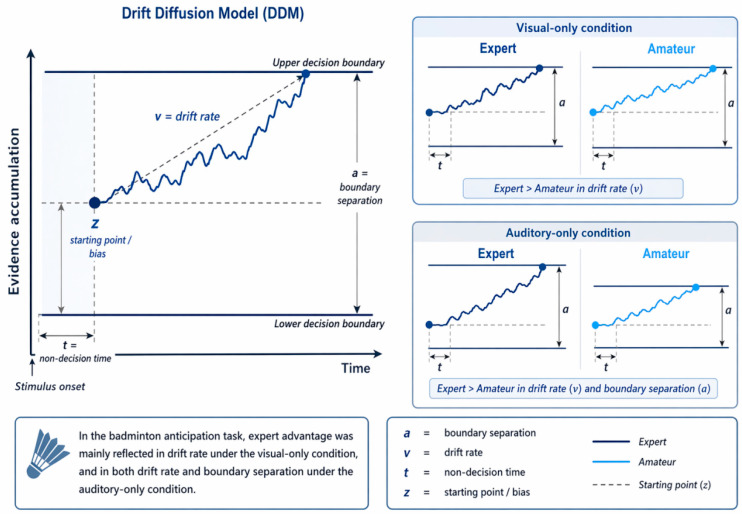
Framework of the drift diffusion model.

Accuracy and reaction time alone are insufficient to fully address these questions. Behavioral indices can indicate who is more accurate and who responds faster, but they cannot determine whether such differences arise from the efficiency of evidence accumulation, decision-threshold settings, non-decision processing time, or starting-point bias. The same behavioral outcomes may even correspond to entirely different internal decision processes (Ratcliff & McKoon, 2008; Ratcliff et al., 2016; He et al., 2025; Weinberg et al., 2025; Yan et al., 2026). The drift diffusion model (DDM) provides an appropriate framework for addressing this issue (see Figure 1). The model can jointly account for accuracy and reaction time in two-choice tasks and decomposes the decision process into the boundary separation parameter a, drift rate v, non-decision time t, and starting-point bias z. Specifically, v reflects the efficiency of evidence accumulation, a reflects decision caution or response threshold, t reflects perceptual encoding and response execution processes, and z reflects bias in the initial decision state. The DDM therefore allows us to examine whether expertise is linked to faster cue-based evidence accumulation, different threshold settings, or changes in non-decision processing (Ratcliff & McKoon, 2008; Ratcliff et al., 2016; Myers et al., 2022). The hierarchical drift diffusion model (HDDM) improves the stability of parameter estimation through hierarchical Bayesian estimation and is particularly suitable for experimental designs involving the joint effects of group and condition (Wiecki et al., 2013).

This study compared anticipatory performance between the expert group and the novice group under visual-only and auditory-only conditions. Accuracy, reaction time, and hierarchical drift diffusion model parameters were jointly analyzed to examine the latent decision-making mechanisms underlying different information channels. HDDM = hierarchical drift diffusion model; *a* = boundary separation parameter; *v* = drift rate; *t* = non-decision time; *z* = starting-point bias.

It should be noted that sport anticipation in real competition is normally supported by temporally coordinated audiovisual information and is closely coupled with action execution. Decoupling perception from action may therefore change not only the response mode but also the perceptual information that performers use. The distinction between vision-for-perception and vision-for-action suggests that perceptual judgments made without action execution may not rely on the same processing pathway as action-guided perception (Goodale & Milner, 1992). In sport-specific anticipation research, gaze behavior and information pickup have also been shown to differ between in situ and video-simulation constraints (Dicks et al., 2010). Therefore, the present study should be understood as examining controlled unimodal perceptual-decision processes rather than audiovisual integration or real-game perception–action coupling.

The present study therefore used a badminton-specific but decoupled perceptual-decision task with two unimodal conditions, namely visual-only and auditory-only conditions. The aim was not to test audiovisual integration or action-coupled anticipation, but to examine how badminton expertise is reflected in behavioral performance and latent decision parameters when visual and auditory information are separately available. Accuracy, reaction time, and hierarchical drift diffusion model parameters were analyzed to determine whether expert advantage under these unimodal cue conditions was mainly associated with evidence accumulation, decision threshold, non-decision time, or starting-point bias.

First, at the behavioral level, the expert group was expected to show higher overall anticipation accuracy than the novice group. This advantage was expected to be observed under both the visual-only and auditory-only conditions, although the magnitude of the expert advantage might differ across sensory channels. Second, regarding condition effects, the visual-only and auditory-only conditions were expected to show different behavioral characteristics, indicating that different sensory channels may support badminton anticipation to different extents. Third, at the level of latent decision-making mechanisms, the expert advantage was expected to be mainly reflected in a higher drift rate *v*, indicating more efficient cue extraction and evidence accumulation. In addition, differences in the boundary separation parameter *a* might also emerge under certain conditions, whereas whether stable group differences exist in non-decision time *t* and starting-point bias *z* remains to be further examined.

## 2. Materials and Methods

### 2.1. Participants

This study adopted a between-group comparative design involving an expert group and a novice group. A total of 66 valid participants were included in the final analysis, with 33 participants in the expert group and 33 in the novice group. Participants in the expert group had received systematic badminton-specific training and had relatively extensive training and competitive experience. Expertise classification was based on badminton-specific training history, competitive experience, and official athlete-grade certification, with reference to the participant-classification framework proposed by McKay et al. (2022). Participants in the novice group had no systematic badminton-specific training experience or only limited general sport experience. All participants had normal or corrected-to-normal vision, normal hearing, and no history of neurological or psychiatric disorders (see Table 1). Before the experiment, all participants provided written informed consent and received either monetary compensation or course credit after completing the experiment. The study protocol was approved by the Ethics Committee of Beijing Sport University, with the ethics approval number 2025376H.

### 2.2. Materials

**Figure 2:**
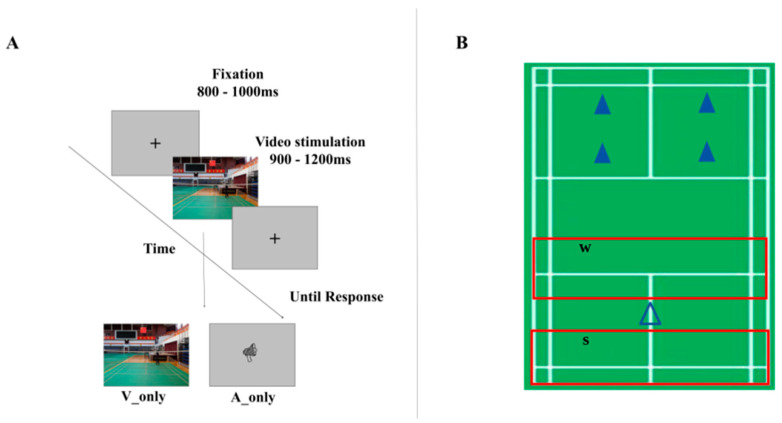
Experimental procedure. (**A**) Each trial began with the presentation of a central fixation cross for 800–1000 ms, followed by the stimulus clip. In the visual-only condition, each visual clip was segmented relative to the moment of racket–shuttle contact and lasted approximately 900–1200 ms. In the auditory-only condition, the corresponding complete racket–shuttle contact sound from the same stroke event was presented. After stimulus presentation, participants were required to press a key as quickly as possible to report their anticipatory judgment regarding the landing region of the shuttlecock, namely upper or lower. (**B**) Task layout and response mapping. Participants viewed the opponent’s return from the perspective indicated by the open triangle. The filled triangles indicate four possible opponent positions, from which the shuttlecock could be directed toward either the upper or lower landing region. The labels W and S indicate the actual response keys used in the experiment: W corresponded to the upper landing region, and S corresponded to the lower landing region. This mapping preserved a vertical, spatially compatible correspondence between the response key and the judged landing region.

The stimuli were video and audio materials from a badminton return anticipation task, designed to assess landing-region anticipation under unimodal information conditions. According to the type of information channel, two experimental conditions were established: Condition B was the visual-only condition, in which participants were presented only with visual cues related to the opponent’s hitting action and the direction of the incoming shuttle; Condition C was the auditory-only condition, in which participants were presented only with auditory cues related to shuttle contact. Participants made an anticipatory judgment about the final landing region of the shuttle based on the available unimodal information (see Figure 2). The stimulus set was based on 120 unique recorded badminton stroke events. For each stroke event, two unimodal stimuli were created: one visual-only stimulus and one auditory-only stimulus. Thus, the formal experiment consisted of 240 trials in total, including 120 visual-only trials and 120 auditory-only trials. No combined audiovisual trials were administered in the present experiment. For each event, the original audiovisual recording was segmented with reference to the moment of racket–shuttle contact. Specifically, the contact frame was defined as the endpoint of the visual clip, and the onset of the visual clip was set approximately 900–1200 ms before this contact moment, depending on the natural temporal structure of the stroke. Thus, the visual-only stimuli contained the preparatory and hitting-action information leading up to racket–shuttle contact, without including extended post-contact shuttle-flight information. The auditory-only stimuli consisted of the corresponding complete racket–shuttle contact sounds extracted from the same recorded stroke events. Thus, the visual-only and auditory-only stimuli were matched at the event level, with both components taken from the same original stroke rather than paired arbitrarily. Because the task required an upper-versus-lower landing-region judgment, the response dimension was defined along the vertical axis rather than the horizontal left–right axis. The visual stimuli were presented at the center of the screen, and no left–right response categories were used. Therefore, any natural left–right variation in the recorded stroke events was not directly mapped onto the response alternatives.

Stimulus presentation and response recording were implemented in MATLAB R2023a (The MathWorks, Inc., Natick, MA, USA) using Psychtoolbox-3 version 3.0.17 on a Lenovo Xiaoxin Pro 16 AHP9 laptop (Lenovo, Beijing, China). Visual stimuli were presented on a 16-inch monitor with a screen resolution of 1920 × 1080 pixels and a refresh rate of 60 Hz. Participants completed the experiment at a viewing distance of approximately 60 cm from the screen. The experimental environment was kept quiet, with stable lighting conditions. Auditory stimuli were presented through in-ear headphones, and the volume was adjusted before the experiment to a level that was clearly discernible and subjectively comfortable for each participant. Keypress responses were recorded using a standard keyboard. The experimental program automatically saved each trial’s response choice, response accuracy, and reaction time.

### 2.3. Procedure

The present study employed a 2 × 2 mixed experimental design. Group was treated as a between-subjects factor, including the expert group and the novice group, whereas condition was treated as a within-subjects factor, including the visual-only and auditory-only conditions. The dependent variables included behavioral indices, namely accuracy and reaction time, as well as latent decision parameters estimated using the hierarchical drift diffusion model.

Before the main experiment, participants first read the experimental instructions and completed practice trials to become familiar with the task procedure and response rules. The practice phase consisted of 16 trials, including eight visual-only trials and eight auditory-only trials. If a participant remained unfamiliar with the task requirements, the experimenter provided additional clarification, but the formal practice session was not repeated. The main experiment consisted of 240 trials, including 120 visual-only trials and 120 auditory-only trials. These trials were divided into three blocks of 80 trials each. Trials from the two unimodal conditions were presented in a randomized or pseudo-randomized order within each block to avoid order effects caused by repeated presentation of the same condition or the same response direction. Short breaks were provided between blocks to reduce fatigue and attentional decline. At the beginning of each trial, a central fixation cross was presented for 800–1000 ms, followed by the experimental stimulus. In visual-only trials, each visual clip lasted approximately 900–1200 ms. This duration was determined with reference to the natural temporal structure of badminton strokes. For each stroke event, the moment of racket–shuttle contact was identified and used as the endpoint of the visual clip. The clip onset was then set approximately 900–1200 ms before this contact moment. A variable rather than fixed duration was used because forecourt and backcourt strokes differed slightly in their preparatory movement and racket-swing duration. This procedure allowed the clips to preserve the key anticipatory information available before and at racket–shuttle contact while avoiding the inclusion of extended post-contact shuttle-flight information.

After stimulus presentation, participants were required to judge as quickly as possible whether the shuttlecock was more likely to land in the upper or lower landing region and to respond by pressing a key.

A spatially compatible key-mapping procedure was used to reduce unnecessary response-selection demands. In the actual task, all participants responded using the W/S keys. The upper landing region corresponded to the W key, whereas the lower landing region corresponded to the S key. Thus, the response mapping preserved a vertical and spatially compatible correspondence between the response key and the judged landing region. No horizontal left–right key mapping was used in the actual task. In addition, response execution was standardized across participants. Participants used the same hand–finger configuration throughout the task, pressing the two response keys with the index and middle fingers of their dominant hand. This configuration was kept constant across the visual-only and auditory-only conditions and across all experimental blocks.

All trials were presented in a randomized or pseudo-randomized order within each block to avoid order effects caused by repeated presentation of the same condition or the same response direction. Before the experiment began, the experimenter explained that the task was to judge the landing region of the incoming shuttle as quickly and accurately as possible based on the currently presented unimodal information, rather than to explicitly report the position of the hitter or details of the hitting action.

### 2.4. Data Analysis

The behavioral data analysis mainly examined differences in accuracy and reaction time across groups and conditions. Accuracy was calculated as each participant’s mean accuracy in the corresponding condition. Reaction time was calculated only for correct-response trials, and each participant’s mean reaction time for correct trials was computed for each condition. Before calculating mean reaction time, trial-level response-time screening was conducted. Trials with missing responses were excluded from reaction-time analysis. In addition, responses faster than 150 ms were treated as anticipatory or accidental responses and were removed. For each participant and condition, reaction times exceeding three standard deviations from that participant’s condition-specific mean were also excluded as outliers. After this screening procedure, mean reaction time was calculated from the remaining correct-response trials for each participant in each condition. The same response-time screening rule was applied before HDDM analysis to reduce the influence of implausible or extreme trial-level reaction times. HDDM analyses were conducted in Python 3.7 using HDDM version 0.9.8 (Wiecki et al., 2013).

Dominant hand was examined as a participant-characteristic variable. Because the number of left-handed participants was small, handedness was not entered as an additional factor in the primary behavioral or HDDM analyses. Instead, the distribution of right- and left-handed participants was compared between the expert and novice groups using Fisher’s exact test.

Before conducting the mixed-design analyses of variance (ANOVAs), the distributions of the dependent variables and the homogeneity of variance were examined. Because the within-subject factor had only two levels, the sphericity assumption was automatically satisfied. Separate 2 (group: expert, novice) × 2 (condition: B visual-only, C auditory-only) mixed-design analyses of variance were then conducted for accuracy and reaction time. Group was entered as a between-subjects factor, and condition was entered as a within-subjects factor. For significant interaction effects, simple effects analyses were conducted. If the interaction effect was not significant, the main effects of group and condition were interpreted. Given that each factor had only two levels, the corresponding pairwise contrast was equivalent to the main-effect test. The reported results included *F* values, degrees of freedom, *p* values, partial *η*^2^, as well as mean differences, 95% confidence intervals, *t* values, and effect sizes for subsequent comparisons.

On the basis of the behavioral data, the present study further introduced hierarchical drift diffusion modeling. This model can integrate accuracy and reaction time information simultaneously and estimate key latent parameters in the decision-making process, including the boundary separation parameter *a*, drift rate *v*, non-decision time *t*, and starting-point bias *z*. Specifically, a reflects the evidence threshold required for an individual to make a decision, v reflects the speed of evidence accumulation, t reflects the time consumed by non-decision processes such as perceptual encoding and response execution, and *z* reflects prior bias in the initial decision state.

During model construction, group and condition were specified as factors influencing latent decision parameters. Six candidate models, from M2 to M7, were established sequentially, and model fit was compared using the Deviance Information Criterion (DIC). The model comparison results indicated that M4 showed the best overall fit. Therefore, subsequent parameter interpretation and statistical inference were based on M4. The specific model was as follows:

M2: depends_on = {v: G × C, t: G × C}
M3: depends_on = {a: G × C, v: G × C, t: G × C}
M4: depends_on = {a: G × C, v: G × C, t: G × C, z: G × C}
M5: M4 + s_v
M6: M5 + s_t
M7: M6 + s_z

After M4 was identified as the optimal model, parameter estimates for each participant under each condition were extracted from M4. Bayesian t-tests were then conducted for *a*, *v*, *t*, and *z* to examine whether significant differences existed between expert players and novice participants. Specifically, differences between the expert group and the novice group were compared for each parameter within each condition. The Bayes factor BF10, mean difference, 95% confidence interval, *t* value, and effect size *d* were reported. A BF10 greater than 3 is generally considered evidence supporting the alternative hypothesis, whereas a BF10 lower than 1/3 is generally considered evidence supporting the null hypothesis. Values between these thresholds indicate that the current evidence is insufficient.

## 3. Results

### 3.1. Behavioral Results

Regarding handedness, 32 experts and 29 novices were right-handed, whereas 1 expert and 4 novices were left-handed. The distribution of dominant hand did not differ significantly between the expert and novice groups, Fisher’s exact test, *p* = 0.355. Therefore, there was no evidence of a systematic between-group imbalance in handedness.

The analysis of variance showed a significant main effect of group, *F*(1, 64) = 56.46, *p* < 0.001, *ηp*^2^ = 0.469, indicating that the expert group showed significantly higher overall accuracy than the novice group. The main effect of condition was also significant, *F*(1, 64) = 12.69, *p* = 0.001, *ηp*^2^ = 0.165, indicating that overall accuracy was higher in the auditory-only condition than in the visual-only condition. The condition × group interaction was not significant, *F*(1, 64) = 0.09, *p* = 0.761, *ηp*^2^ = 0.001.

Because the condition × group interaction was not significant, the main effects of group and condition were interpreted. Given that each factor had only two levels, the corresponding pairwise contrast was equivalent to the main-effect test. The results showed that, after collapsing across the two groups, accuracy in condition C was significantly higher than that in condition B, mean difference = 0.068, 95% CI [0.030, 0.106], *t*(65) = 3.59, *p* = 0.001, *dz* = 0.442. After collapsing across the two conditions, the expert group showed significantly higher overall accuracy than the novice group, mean difference = 0.148, 95% CI [0.108, 0.187], *t*(64) = 7.51, *p* < 0.001, *d* = 1.850.

The reaction time results were as follows. In the visual-only condition B, the mean reaction time was 2200.04 ms for the expert group, SD = 222.23, and 2228.45 ms for the novice group, SD = 340.92. In the auditory-only condition C, the mean reaction time was 2512.98 ms for the expert group, SD = 195.00, and 2528.87 ms for the novice group, SD = 229.98.

The 2 × 2 mixed-design analysis of variance showed a significant main effect of condition, *F*(1, 64) = 150.32, *p* < 0.001, *ηp*^2^ = 0.701, indicating that reaction times were significantly longer in the auditory-only condition than in the visual-only condition. The main effect of group was not significant, *F*(1, 64) = 0.15, *p* = 0.699, *ηp*^2^ = 0.002, indicating no significant overall difference in reaction time between expert players and novice participants. The condition × group interaction was also not significant, *F*(1, 64) = 0.06, *p* = 0.803, *ηp*^2^ = 0.001.

**Figure 3:**
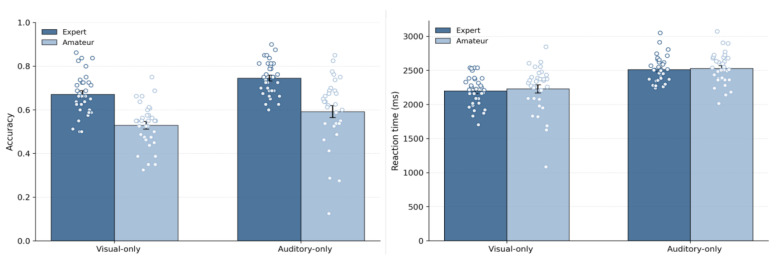
Behavioral performance of the expert group and novice group under the visual-only and auditory-only conditions.

Because the condition × group interaction was not significant, the main effects of group and condition were interpreted. Given that each factor had only two levels, the corresponding pairwise contrast was equivalent to the main-effect test. The results showed that, after collapsing across the two groups, reaction times in condition C were significantly longer than those in condition B, mean difference = 306.68 ms, 95% CI [257.09, 356.27], *t*(65) = 12.35, *p* < 0.001, *dz* = 1.520. After collapsing across the two conditions, the overall difference in reaction time between the expert group and the novice group was not significant, mean difference = −22.15 ms, 95% CI [−136.23, 91.93], *t*(64) = −0.39, *p* = 0.699, *d* = −0.095 (see Figure 3).

The left panel shows accuracy, and the right panel shows reaction time for correct responses. Bars represent the mean values for each group under each condition, error bars represent standard errors, and scatter points represent individual participants’ mean values.

### 3.2. HDDM

Between-group comparisons of HDDM parameters were conducted separately under the visual-only and auditory-only conditions. The difference direction was defined as expert group minus novice group. For each comparison, Bayes factor, mean difference, 95% confidence interval, *t* value, *p* value, and Cohen’s *d* were reported.

Under the visual-only condition, there was very strong evidence for a between-group difference in drift rate v between expert players and novice participants, BF10 = 1992.845. The expert group showed a significantly higher v than the novice group, with a mean difference of 0.281, 95% CI [0.165, 0.397], *t*(64) = 4.846, *p* < 0.001, *d* = 1.193. This indicates that, under the visual-only condition, expert players demonstrated greater efficiency of evidence accumulation than novice participants. In contrast, there was no strong evidence for between-group differences in boundary separation a, non-decision time t, or starting-point bias z. Specifically, for boundary separation a, BF10 = 0.358, mean difference = 0.014, 95% CI [−0.045, 0.073], *t*(64) = 0.480, *p* = 0.633, *d* = 0.118. For non-decision time t, BF10 = 0.299, mean difference = 0.026, 95% CI [−0.076, 0.128], *t*(64) = 0.505, *p* = 0.615, *d* = 0.124. For starting-point bias *z*, BF10 = 0.259, mean difference = 0.016, 95% CI [−0.001, 0.033], *t*(64) = 1.834, *p* = 0.071, *d* = 0.451.

Under the auditory-only condition, there was clear evidence for between-group differences in both boundary separation a and drift rate v between expert players and novice participants. The expert group showed a higher boundary separation parameter a than the novice group, BF10 = 53.095, mean difference = 0.108, 95% CI [0.049, 0.167], *t*(64) = 3.652, *p* < 0.001, *d* = 0.899. The expert group also showed a higher drift rate *v* than the novice group, BF10 = 24.354, mean difference = 0.228, 95% CI [0.092, 0.363], *t*(64) = 3.359, *p* = 0.001, *d* = 0.827. These findings suggest that, under the auditory-only condition, expert players not only exhibited greater efficiency of evidence accumulation but also showed greater decision caution or a higher response threshold. In contrast, there was no strong evidence for a between-group difference in non-decision time t, BF10 = 0.297, mean difference = −0.053, 95% CI [−0.121, 0.015], *t*(64) = −1.560, *p* = 0.124, *d* = −0.384. For starting-point bias *z*, BF10 = 0.426, mean difference = 0.019, 95% CI [0.001, 0.038], *t*(64) = 2.066, *p* = 0.043, *d* = 0.509. Although the frequentist test reached statistical significance for *z*, the Bayes factor indicated that the current evidence remained insufficient; therefore, this result should be interpreted cautiously.

**Figure 4:**
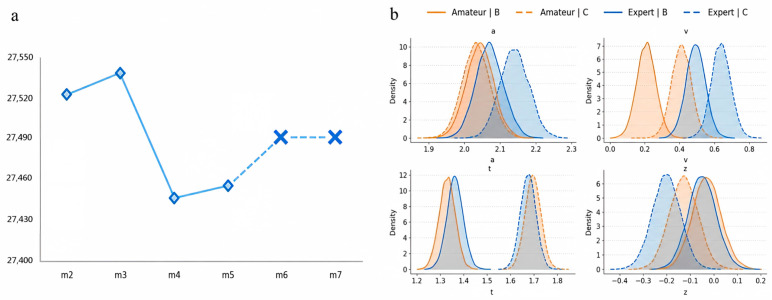
Comparison of HDDM candidate models and posterior distributions of key parameters in the optimal model. (**a**) Model comparison results for candidate models M2 to M7. M4 showed the lowest model comparison index, indicating the best model fit; therefore, it was selected as the optimal model for subsequent parameter interpretation and statistical testing. In panel (**a**), diamond markers are used for M2–M5, whereas cross markers are used for M6–M7; the marker and line styles are used only for visual differentiation and do not indicate different statistical categories. (**b**) Posterior distributions of key parameters in M4 for the expert group and novice group under the visual-only condition B and auditory-only condition C. The distributions are shown for the boundary separation parameter *a*, starting-point bias *z*, non-decision time *t*, and drift rate *v*. Different colors and line types indicate different groups and conditions.

Overall, the HDDM results showed that expert players had higher drift rates than novice participants under both visual-only and auditory-only conditions. The model-comparison results and posterior distributions of the key parameters are presented in Figure 4. In addition, under the auditory-only condition, experts also showed higher boundary separation. No robust evidence was found for stable group differences in non-decision time or starting-point bias.

## 4. Discussion

The present study compared expert and novice performance in a badminton anticipation task under visual-only and auditory-only conditions, and used HDDM to examine possible group differences in latent decision processes. The results showed that the expert group demonstrated significantly higher overall accuracy than the novice group, whereas no significant group difference was observed in overall reaction time. In addition, the auditory-only condition showed higher accuracy than the visual-only condition, but it was also accompanied by longer reaction times. The HDDM results indicated that, in the visual-only condition, the expert advantage was mainly reflected in a higher drift rate *v*, whereas in the auditory-only condition, the expert advantage was reflected in both a higher drift rate v and a higher boundary separation parameter a. Although the surface-level behavioral results did not show a significant group × condition interaction, the latent decision parameters may indicate a channel-specific processing pattern. Overall, the findings support the main hypotheses and suggest that expert advantage is reflected in both behavioral performance and model-estimated decision processes. However, these findings should be interpreted within the constraints of a controlled unimodal video-/audio-based button-press task, rather than as direct evidence of audiovisual integration or action-coupled anticipation in real badminton performance.

From the behavioral results, the expert group showed significantly higher overall accuracy than the novice group, whereas no significant group difference was found in overall reaction time. This pattern suggests that the expert advantage lay mainly in more accurate outcome judgments without an additional time cost. This is consistent with the general conclusions of research on perceptual–cognitive expertise in sport. Compared with novices, experts are often not merely faster responders; rather, they may be able to extract, select, and use key information more effectively within a comparable time window, thereby producing more accurate judgments (Mann et al., 2007; Müller & Abernethy, 2012). In studies of naturalistic decision making in badminton, experts have also been found to organize judgment processes more effectively by integrating kinematic and contextual cues, enabling them to maintain high prediction accuracy under substantial time pressure (Macquet & Fleurance, 2007; De Waelle et al., 2023).

Regarding the condition effect, the present study found that accuracy was higher in the auditory-only condition than in the visual-only condition, whereas reaction times were also significantly longer in the auditory-only condition. This suggests that the two unimodal sources of information may have different advantages and limitations in supporting badminton anticipation. Auditory cues have high temporal resolution and can provide fine-grained information at the moment of shuttle contact, including cues related to hitting force, contact quality, and rhythm changes. Such information may therefore enhance the reliability of outcome judgments (Sors et al., 2017; Hülsdünker et al., 2021). However, auditory cues are weaker than visual cues in conveying spatial structure and movement trajectory. Participants may need to further transform transient sound information into an inference about the spatial outcome of the landing region, and this process may increase the time required for evidence integration and decision completion. In the present task, auditory information may therefore be characterized by high temporal precision but relatively low spatial directness, resulting in a behavioral pattern of higher accuracy but slower responses (Sors et al., 2017; X. Wang et al., 2023). This indicates that, in unimodal badminton anticipation, information effectiveness and response speed do not necessarily change in parallel.

At the behavioral level, no significant group × condition interaction was observed. Based on surface-level behavioral indices, expert players and novice participants showed broadly similar condition-related trends across the two unimodal conditions. However, this does not mean that the two groups relied on the same internal processing mechanisms. Research using diffusion models has long suggested that similar combinations of accuracy and reaction time can arise from different underlying parameter structures. Therefore, when the research question concerns the mechanisms through which expert advantage is achieved, relying only on behavioral means is often insufficient (Ratcliff & McKoon, 2008; Ratcliff et al., 2016). The subsequent HDDM results of the present study are consistent with this view.

In the visual-only condition, the difference between expert players and novice participants was mainly reflected in drift rate *v*, whereas boundary separation parameter a, non-decision time *t*, and starting-point bias *z* did not show stable group differences. According to the diffusion model framework, *v* is commonly interpreted as reflecting the efficiency of evidence accumulation per unit time. Therefore, the higher v observed in expert players under the visual-only condition indicates that experts may be able to extract useful information from visual cues more rapidly and transform it into evidence supporting the decision. This result is highly consistent with classic models of expert advantage in striking sports, which propose that one of the core advantages of experts lies in their more efficient use of body posture, racket-swing trajectory, and kinematic cues before and after contact, thereby enabling faster and more stable outcome predictions (Abernethy & Russell, 1987; Abernethy et al., 2001; Müller & Abernethy, 2012; Gredin et al., 2023; D. Wang et al., 2025; Wu et al., 2026). The present findings suggest that, under the visual-only condition, expert advantage is not primarily reflected in greater decision caution, nor does it mainly arise from faster perceptual encoding or motor execution. Instead, it is more likely to reflect optimization of visual information extraction and evidence accumulation. This provides additional computational modeling evidence for expert visual advantage (Ratcliff & McKoon, 2008; Myers et al., 2022).

Different from the visual-only condition, in the auditory-only condition, expert players showed not only a higher drift rate v but also a higher boundary separation parameter a. The higher v suggests that experts also used auditory cues more efficiently. Auditory anticipation therefore appears to be more than an auxiliary form of visual anticipation; it may be another channel through which sport-specific experience shapes performance. Previous studies have shown that hitting sounds, sound timing, and audio–visual congruence can significantly affect force judgment, trajectory expectation, and response preparation in ball sports, indicating that auditory information itself may contain substantial sport-specific information (Sors et al., 2017; Hülsdünker et al., 2021; X. Wang et al., 2023; X. Wang et al., 2025). The present study further suggests that expert advantage in this channel is also first reflected in improved efficiency of evidence accumulation.

The expert group also showed a higher boundary separation parameter a in the auditory-only condition. Within the diffusion model framework, a is commonly interpreted as the amount of evidence required to reach a decision; a higher a generally indicates a more cautious and robust decision strategy (Ratcliff & McKoon, 2008; Ratcliff et al., 2016). Considering the characteristics of the present task, this result may indicate that, under the auditory-only condition, experts were not simply faster at interpreting auditory cues. Instead, they appeared to combine efficient auditory evidence accumulation with a more cautious evidence criterion. Because auditory cues have high temporal precision but are relatively indirect in mapping onto spatial landing outcomes, experts may have developed more mature judgment strategies through long-term training. When facing this type of unimodal information, they may both accumulate evidence effectively and maintain a higher evidence threshold before responding, thereby reducing the risk of premature decisions and incorrect judgments. Thus, expert advantage in the auditory-only condition was reflected not only in faster processing but also in more stable decision making, suggesting a more complex mechanism structure than that observed in the visual-only condition.

By contrast, the present study did not find stable group differences in t under either condition, and the evidence for *z* was also generally weak. This suggests that the key differences between expert players and novice participants were not mainly reflected in the speed of perceptual encoding and response execution, nor in a strong prior response bias. Instead, they were concentrated in the efficiency of evidence accumulation and, in the auditory-only condition, in an additional difference in decision threshold. This pattern is theoretically informative. It indicates that expert advantage is not a general phenomenon of simply faster processing or better responding, but rather a differentiated combination of several latent decision components across different sensory channels. Among these components, drift rate v may represent a relatively stable core index of expert advantage across channels, whereas boundary separation parameter a may reflect a strategic regulatory component that is activated under specific channels or task demands (Ratcliff & McKoon, 2008; Ratcliff et al., 2016; Myers et al., 2022).

From the perspective of the research hypotheses, the present findings generally support the hypothesis that the expert group would show a higher level of anticipation performance at the behavioral level. They also support the hypothesis that expert advantage would be mainly reflected in drift rate *v*. At the same time, the results indicate that expert advantage is not completely homogeneous across different channels. Although no significant interaction was observed at the behavioral level, HDDM revealed a differentiated structure in which expert advantage under the visual-only condition was mainly reflected in the efficiency of evidence accumulation, whereas expert advantage under the auditory-only condition involved both greater efficiency of evidence accumulation and greater decision caution. This suggests that expert advantage under unimodal conditions has a certain degree of channel specificity, and that such specificity can only be clearly identified after behavioral outcomes are decomposed into latent parameters. This supports the use of HDDM in sport anticipation research because it helps link behavioral differences to possible differences in underlying decision processes (Wiecki et al., 2013; Myers et al., 2022).

The present findings offer preliminary implications for controlled perceptual–cognitive training under unimodal cue conditions. For example, laboratory-based visual tasks may be used to explore the extraction of kinematic and early trajectory-related information, whereas auditory tasks may be used to explore the interpretation of contact sounds when spatial information is limited. However, these possibilities should not yet be treated as established training prescriptions. In particular, the present button-press findings do not demonstrate that training cue extraction or decision thresholds under decoupled task constraints will improve on-court performance. Their application to representative, action-coupled badminton training therefore requires further validation (Hülsdünker et al., 2021; X. Wang et al., 2023).

Several limitations should be acknowledged.

First, the absence of a combined audiovisual condition is an important methodological limitation. Establishing the temporal correspondence between visible racket–shuttle contact and impact sound is a prerequisite for constructing valid audiovisual stimuli. Because no such condition was administered, the present findings cannot determine whether expert advantage would be enhanced, reduced, or reorganized when visual and auditory cues are presented together. The results therefore provide no evidence concerning audiovisual integration, audiovisual congruency, or natural multisensory anticipation in badminton and should be restricted to separate visual-only and auditory-only perceptual-decision processes. Future work could address this limitation by using frame-level validated audiovisual alignment and directly comparing visual-only, auditory-only, and audiovisual conditions.

The present study drew mainly on behavioral data and HDDM parameters and did not include eye-tracking, electroencephalographic, or kinematic measures (D. Wang et al., 2025; Yan et al., 2026). Therefore, direct evidence regarding the neural temporal processes and cue-extraction pathways underlying expert advantage remains limited.

Although the stimuli were derived from badminton-specific stroke events, the task was a video-/audio-based button-press task and therefore decoupled perception from action execution. This issue is not merely a limitation of ecological scope. Theoretical work on vision-for-perception and vision-for-action suggests that perceptual judgments made without action execution may involve processing mechanisms that differ qualitatively from those involved in action-guided perception (Goodale & Milner, 1992). Consistent with this view, sport-specific evidence has shown that gaze behavior and information pickup can differ systematically between in situ and video-simulation task constraints (Dicks et al., 2010). Therefore, the present findings should not be generalized directly to anticipation as it operates in actual badminton performance. More precisely, the current task assesses a controlled perceptual-decision component of badminton anticipation under decoupled task constraints. Future research should combine unimodal and audiovisual stimuli with more representative action-coupled designs, such as in situ responses, sport-specific motor responses, virtual reality, or eye-tracking measures, to determine whether the present HDDM-based decision parameters also hold under more representative perception–action coupling.

In addition, the use of the standard DDM/HDDM framework should be interpreted within the constraints of the present experimental design. The model assumes evidence accumulation toward relatively fixed decision boundaries and does not incorporate an explicit urgency-to-act mechanism or parallel specification of competing action plans (Cisek, 2007; Cisek et al., 2009). These assumptions do not fully capture the continuous and dynamically time-constrained nature of on-court decision making in fast interceptive sports. Therefore, the boundary separation parameter observed in the present study should not be interpreted as a complete representation of dynamic response thresholds in real competition (Cisek et al., 2009; Beck et al., 2026). Rather, in the context of the present two-choice temporal-occlusion task, it should be understood as an index of the amount of evidence required before making a discrete landing-region judgment.

Future studies should combine temporal-occlusion paradigms with more representative response modes, such as movement initiation, court-positioning responses, or virtual-reality-based interception tasks, to examine whether the present decision-parameter patterns generalize to more action-coupled badminton contexts (Avilés et al., 2019; Beck et al., 2026). Future studies could further combine neurophysiological indices, trial-level modeling, and true combined audiovisual conditions to examine how visual and auditory cues are weighted or combined when presented together.

## 5. Conclusions

The expert group showed significantly higher overall accuracy than the novice group, whereas the difference in overall reaction time was not significant. In addition, the auditory-only condition produced higher accuracy than the visual-only condition, but this was accompanied by longer reaction times. The expert advantage in unimodal anticipation was therefore reflected not only in behavioral performance but also in latent decision parameters. Specifically, drift rate v appeared to be the more stable marker of expert advantage across the two unimodal conditions, whereas boundary separation a showed a condition-specific pattern. These conclusions are limited to the present controlled unimodal video-/audio-based button-press perceptual-decision task and should not be interpreted as evidence of audiovisual integration, audiovisual congruency, natural multisensory anticipation, or action-coupled anticipation in real badminton performance.

## Figures and Tables

**Table 1:** Participant characteristics.

Variable	Expert Group (*n* = 33)	Novice Group (*n* = 33)	Statistic	*p*
Age, years	22.00 ± 2.70	21.85 ± 2.44	*t* = 0.239	0.812
Sex, male/female	16/17	18/15	χ^2^ ≈ 0.243	0.622
Training experience, years	15.15 ± 2.97	0.73 ± 0.80	*t* = 26.937	<0.001
Dominant hand, right/left	32/1	29/4	Fisher’s exact test	0.355
Athlete grade				
Master-level athlete	1	0		
First-class athlete	13	0		
Second-class athlete	19	0		
No athlete grade certificate	0	33		

## Data Availability

The de-identified behavioral data and analysis scripts supporting the findings of this study are available from the corresponding author upon reasonable request due to ethical restrictions.
